# Managing substance abuse on psychiatric units: a scoping review

**DOI:** 10.3389/fpsyt.2025.1653093

**Published:** 2025-10-29

**Authors:** Alexandre Hudon, Jean-Philippe Cloutier-Tanguay, Joshua Levy, William Dastous-Stampe, Marie-Louise Daigneault, Cédric Lacombe, François Noël, Stéphanie Borduas Pagé

**Affiliations:** ^1^ Department of Psychiatry and Addictology, Faculty of Medicine, Université de Montréal, Montréal, QC, Canada; ^2^ Department of Psychiatry, Institut Universitaire en Santé Mentale de Montréal, Montréal, QC, Canada; ^3^ Centre de Recherche de l’Institut Universitaire en Santé Mentale de Montréal, Montréal, QC, Canada; ^4^ Department of Psychiatry, Institut National de Psychiatrie Légale Philippe-Pinel, Montréal, QC, Canada; ^5^ Groupe Interdisciplinaire de Recherche sur la Cognition et le Raisonnement Professionnel (GIRCoPRo), Université de Montréal, Montréal, QC, Canada; ^6^ Department of Psychiatry, Faculty of Medicine, Université Laval, Québec, QC, Canada; ^7^ Department of Family and Emergency Medicine, Faculty of Medicine, Université de Montréal, Montréal, QC, Canada

**Keywords:** psychiatric inpatient, substance use, management, drug screening, harm reduction, brief intervention, policy, dual diagnosis

## Abstract

**Objective:**

Substance use during psychiatric hospitalization compromises safety, treatment engagement, and post-discharge outcomes, but practical guidance for ward staff remains limited. This scoping review mapped the peer-reviewed literature on how psychiatric inpatient units detect, manage, and respond to alcohol or drug use that occurs on the ward itself, and summarized the effectiveness and breadth of reported strategies.

**Methods:**

The review followed the PRISMA-ScR framework. PubMed, Embase, PsycINFO, and Google Scholar were searched from inception to April 2025 using controlled vocabulary and free-text terms for substance use, psychiatric inpatients, and management strategies. English- and French-language empirical studies, quality-improvement reports, policy evaluations, and scoping reviews were eligible if they described an intervention or protocol applied in an inpatient psychiatric setting. Reviewers independently screened titles/abstracts and full texts extracted data with a standardized spreadsheet, and applied Joanna Briggs Institute critical-appraisal tools.

**Results:**

From the identified studies, 18 studies met inclusion criteria: 1 randomized trial, 3 quasi-experimental reports, 8 descriptive prevalence/cross-sectional studies, 2 policy case studies, 3 reviews/chapters, and 1 commentary. Seven recurring intervention domains were identified: systematic screening (n = 9 studies), brief motivational interventions, policy / protocol development, environmental or security measures, harm reduction strategies, staff training and culture change, and discharge planning. Structured screening improved detection rates up to two-fold; brief interventions such as SBIRT and BIMI increased post-discharge treatment engagement and reduced 30-day readmissions by up to 18%. Zero-tolerance security measures showed inconsistent effects on contraband entry or aggression. Overall methodological quality was moderate, with most evidence derived from single-site implementations.

**Conclusions:**

Existing evidence suggests that standardized screening, ward-adapted brief interventions, clear patient-centered policies, and targeted harm-reduction measures can feasibly improve management of inpatient substance use, while purely punitive security tactics are insufficient. Research gaps include rigorous multi-site evaluations, adolescent and forensic settings, and integrated harm-reduction protocols for substances other than nicotine.

## 1 Introduction

Substance use and mental health disorders intersect in complex, bidirectional ways that challenge clinicians and policymakers alike. Large epidemiologic surveys show that mood or anxiety disorders approximately double the odds of meeting criteria for a substance-use disorder, underscoring the scale of the problem across community samples (1, 2). Recent work in neurosciences suggests that overlapping neurobiological circuits (particularly those governing reward salience and stress responsivity) create shared vulnerability, helping to explain why nearly half of adults entering treatment for addiction also meet criteria for another psychiatric diagnosis (3). Longitudinal data indicate that early-onset internalizing conditions markedly elevate later risk for nicotine, alcohol, and illicit-drug dependence, even after controlling for sociodemographic factors (4). Alcohol illustrates the bidirectionality: heavy drinking can precipitate depressive episodes while existing depression predicts escalation from hazardous to dependent use (5). The cannabis–psychosis link is similarly reciprocal; frequent high-potency consumption accelerates transition to first-episode psychosis, but psychotic disorders themselves are associated with higher rates of continued cannabis use and relapse (6). Comorbidity also complicates clinical trajectories: benzodiazepine misuse in anxiety or post-traumatic stress disorders is associated with poorer functional outcomes and greater healthcare utilization while co-occurring alcohol or drug misuse predicts lower adherence to antidepressants and mood stabilizers (7, 8). Nonetheless, recovery research highlights protective factors, often termed “recovery capital”, that buffer relapse and psychiatric recurrence, including stable housing, social support, and engagement in mutual-help groups (9). Harm-reduction frameworks further emphasize that partial improvements (such as nicotine-replacement therapy for smokers with schizophrenia) can yield measurable gains in cognition and quality of life even when abstinence is not immediately attainable (10). Contemporary translational models therefore call for integrated, stage-matched interventions that address neurocognitive deficits, social disadvantage, and psychiatric symptom burden in tandem (11).

International guidelines increasingly endorse a multifaceted approach to substance-use disorders (SUDs), combining evidence-based pharmacotherapies with psychosocial and harm-reduction interventions across the continuum of care (12–14). For opioid, alcohol, and nicotine use disorders, first-line treatments and preventive strategies are now well established in community and outpatient settings (15, 16). However, much less guidance exists on how to adapt these interventions for psychiatric inpatient units, where patients may be acutely unwell, pharmacologic regimens are often interrupted, and patterns of substance use differ. Screening and brief interventions are promoted as preventive services, but their implementation on psychiatric wards remains inconsistent, and few studies have addressed how best to manage co-occurring psychiatric and substance-use disorders during hospitalization itself (17). This demonstrates the need for unit-level protocols that can bridge evidence-based addiction care with the realities of acute psychiatric treatment.

Although inpatient psychiatric wards routinely report that between one-quarter and one-half of service users drink alcohol or use illicit drugs while admitted, authoritative guidance on how staff should respond remains strikingly sparse (18). National frameworks for SUDs (such as the Royal College of Psychiatrists’ quality standards or WHO’s Mental Health Gap Action Programme) devote only brief sidebars to acute-care settings and focus primarily on discharge planning rather than real-time use on the ward (19, 20). Most institutions therefore default to zero-tolerance rules that rely on random searches, yet observational audits show these measures neither prevent entry of substances nor reduce subsequent aggression (21, 22). Brief-intervention models such as Screening, Brief Intervention, and Referral to Treatment (SBIRT) have been tested in emergency departments but have not been adapted to locked or restricted-entry wards, leaving uncertainty about staffing ratios, confidentiality, and capacity to obtain informed consent from acutely unwell patients (23, 24). Similarly, contingency-management approaches have never been evaluated behind ward doors, largely due to ethical concerns about “rewarding” patients in coercive environments (25). In the absence of evidence-based direction, nurses report using *ad-hoc* strategies that vary by shift and rely on personal comfort levels, contributing to inconsistent practice and patient perceptions of arbitrariness (26). This gap highlights a need for consensus-driven protocols and high-quality implementation studies that address the unique legal, ethical, and clinical complexities of concurrent substance use and mental health problems occurring inside psychiatric hospitals.

This scoping review aims to identify the existing evidence on how psychiatric inpatient wards recognize and handle alcohol or drug use that occurs during admission. Specifically, the review will describe the types of strategies and note where these have been applied and what outcomes were recorded. A secondary objective is to outline practical, low-burden recommendations that emerge from the literature. These suggestions are intended as starting points for local quality-improvement efforts and to highlight areas where more focused research would be valuable.

## 2 Methods

### 2.1 Search strategies

A comprehensive search strategy was developed to identify studies addressing the management of substance use within psychiatric inpatient settings. Four electronic databases were searched from their inception through April 2025: PubMed (MEDLINE), Embase, PsycINFO, and the Google Scholar search engine. Search strategies combined controlled vocabulary terms (e.g., MeSH, Emtree, APA Thesaurus) with free-text keywords related to substance use and misuse (e.g., “substance use disorder,” “alcohol abuse,” “drug misuse”), psychiatric inpatient care (e.g., “psychiatric ward,” “mental health inpatient,” “locked unit”), and management or intervention strategies (e.g., “treatment,” “SBIRT,” “harm reduction,” “policy,” “discharge planning”). Boolean operators were used to combine concepts across the three core domains, and additional syntax refinements were applied in each database to optimize retrieval. The search strategy was iteratively refined in consultation with a health sciences librarian specialized in psychiatry and addiction medicine. No geographic or setting restrictions were applied. Only studies published in English or French were eligible. Reference lists of included articles and relevant reviews were manually screened to identify additional studies. The final search results were de-duplicated and screened in Rayyan by two reviewers independently, with discrepancies resolved through consensus. The complete database-specific search strings are presented in **Supplementary Material**. The Preferred Reporting Items for Systematic reviews and Meta-Analyses adapted for Scoping Review checklist (PRISMA-SRc) is also found in the provided **Supplementary Material**. This study was not registered.

### 2.2 Study eligibility

Studies were considered eligible if they examined the detection, management, intervention, or policy response to substance use or SUDs within psychiatric inpatient settings. Eligible settings included general adult psychiatric wards, adolescent psychiatric units, forensic psychiatric hospitals, emergency or brief-stay psychiatric beds, and specialized mental health in-patient programs. Also, eligible study designs included empirical research (quantitative, qualitative, or mixed-methods), quality-improvement projects, implementation studies, clinical audits, policy evaluations, and scoping reviews. To be included, studies needed to report on specific management strategies, such as structured screening (e.g., urine toxicology, drug screening questionnaires), brief interventions (e.g., SBIRT), harm-reduction measures (e.g., e-cigarettes, take-home naloxone), discharge planning, staff training, or unit-level policy implementation. Articles were required to include information on intervention design, implementation process, reported outcomes, or contextual barriers and facilitators. Considering the small amounts of studies on the topic, commentaries and perspectives were also included. Only publications available in English or French and published from inception of the databases onward were included.

Exclusion criteria encompassed studies focused exclusively on outpatient, community-based, or emergency department populations, unless psychiatric inpatients were explicitly included. Articles that described substance use solely as a background risk factor (without addressing detection or management) were excluded. Studies conducted in non-psychiatric hospital settings (e.g., internal medicine or surgical wards) were excluded unless they involved embedded psychiatric services. Other exclusions included single case reports, and papers without available full text. These criteria were established prior to screening and applied consistently during the title/abstract and full-text review phases.

### 2.3 Data extraction

Data extraction was performed using a structured Excel spreadsheet (Microsoft 365 version). For each included study, the following variables were charted (1): Author(s), year, and country (2), Sample population, including setting, sample size, and clinical context (3); Type of psychiatric inpatient unit (e.g., general adult, adolescent, forensic, brief-stay) (4); Substances examined, detailing the types of substance use addressed (e.g., alcohol, cannabis, opioids, nicotine) (5); Description of how substance use was addressed, including management strategies, screening tools, brief interventions, harm-reduction approaches, and institutional policies (6); Description of how substance use was problematic, as reported by the study (e.g., interference with care, safety concerns, diagnostic challenges) (7); Main outcomes, such as changes in detection rates, treatment engagement, readmissions, or policy impact; and (8) Main conclusions or implications drawn by the authors. Data extraction was conducted by one reviewer and verified by a second to ensure completeness and accuracy. Discrepancies were resolved through discussion. Discrepancies at both the title/abstract and full-text screening stages were resolved through discussion between the two primary reviewers. If consensus could not be reached, a third reviewer was consulted to adjudicate. This same process was applied during data extraction and quality appraisal to ensure accuracy and consistency.

### 2.4 Data analysis

During the synthesis phase, the research team conducted an inductive thematic grouping of the extracted interventions and policies. Through iterative review and consensus discussions, seven core domains were identified that reflect the principal areas of ward-level practice described in the literature (screening, brief interventions, policy frameworks, environmental and security measures, harm reduction, smoke-free strategies, and staff training/continuity of care). These domains were used to structure the summary table and guide the narrative synthesis.

### 2.5 Quality assessment

A structured quality appraisal was conducted to provide an overview of the methodological aspects and transparency of included studies. Each study was assessed using the Joanna Briggs Institute (JBI) critical appraisal checklists, selected based on study design (27). The JBI tools for randomized controlled trials, quasi-experimental studies, prevalence studies, qualitative research, text and opinion papers, and scoping reviews were applied as appropriate. Quality appraisal focused on elements such as clarity of research objectives, sampling procedures, validity of measurement tools, transparency in intervention description, and appropriateness of analytical methods. Two reviewers independently assessed each study, with disagreements resolved through discussion and consensus. No studies were excluded based on quality assessment; rather, appraisal findings were used to contextualize the strength and consistency of the evidence base across study types.

## 3 Results

### 3.1 Description of the identified studies

**Figure 1:**
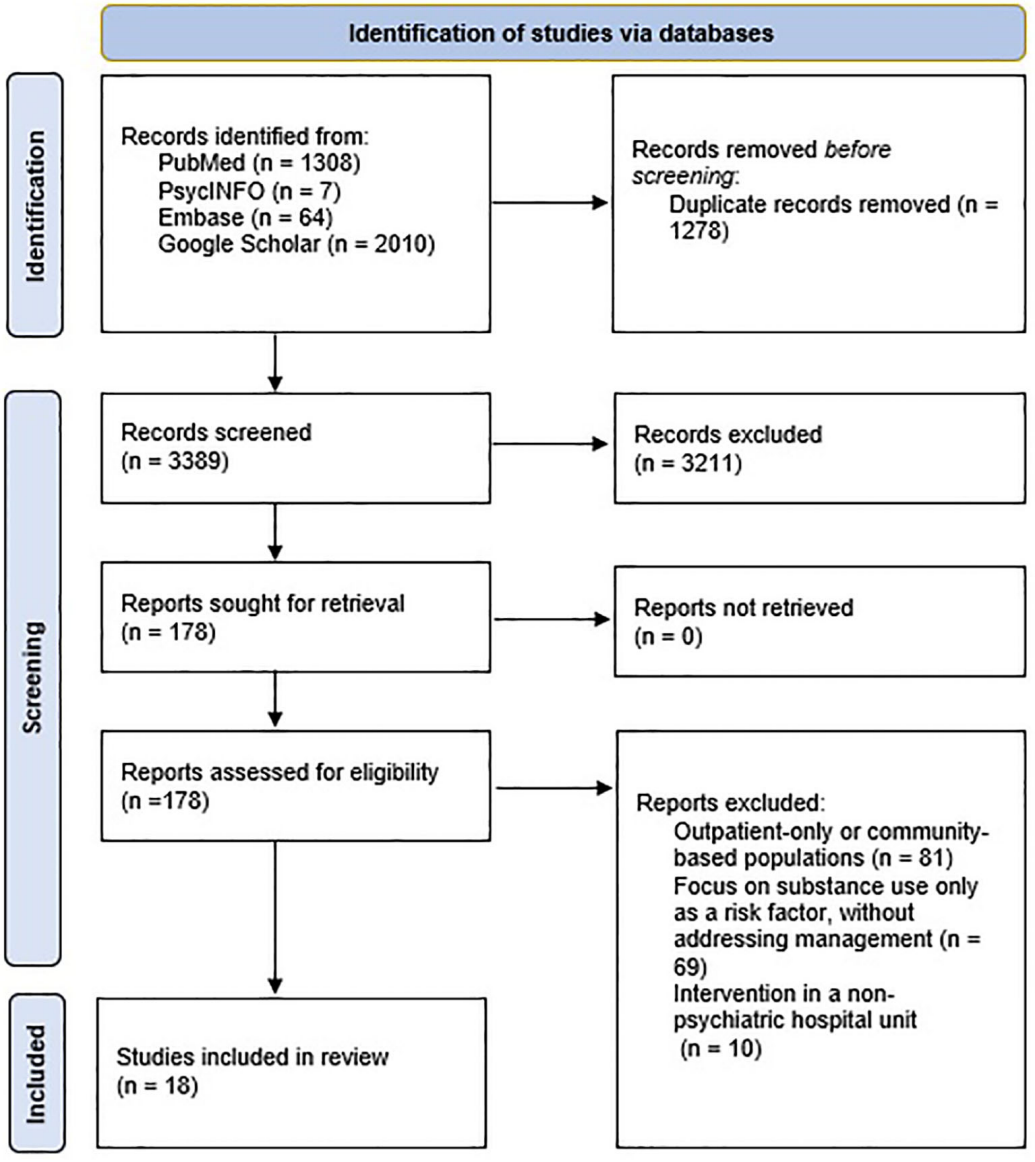
Flow diagram for the identification of studies.

This scoping review examined published literature on the management of substance use within psychiatric inpatient units. A total of 4389 records were identified through comprehensive database searching, including PubMed (n = 1308), PsycINFO (n = 7), Embase (n = 64), and Google Scholar (n = 2010). After removing 1278 duplicates, 3389 unique records were screened by title and abstract. Of these, 3211 were excluded for not meeting the inclusion criteria. Full-text assessment was conducted for 178 studies. Following detailed review, 160 articles were excluded for the following reasons: focus on outpatient or community-based populations (n = 81), discussion of substance use only as a risk factor without management components (n = 69), or focus on interventions outside of psychiatric hospital settings (n = 10). Ultimately, 18 studies were included for analysis. The complete PRISMA flow diagram is presented in **Figure 1**. These studies represent a diverse body of literature, including randomized trials, quality-improvement reports, prevalence surveys, and hospital policy evaluations. Notably, most interventions were implemented in general adult psychiatric wards, although a few targeted adolescent or specialized brief-stay units. Approaches to managing substance use varied widely, including structured screening (e.g., urine toxicology, questionnaires), motivational interventions, policy reforms, and harm-reduction practices. **Table 1** provides a detailed overview of each included study.

**Table 1:** Identified studies and detailed analysis.

Study #	Authors (year, country)	Sample population	Type of unit	Substances examined	How substance use was addressed	How substance use was problematic	Main outcomes	Main conclusions	Quality Assessment
1	Prochaska et al., 2005 (USA) (30)	22–bed adult psychiatry ward; 250 consecutive discharges pre–change (1998–2001) plus 78 substance–misusing inpatients post–change	General adult acute inpatient ward, university teaching hospital	Alcohol; cannabis; stimulants; opioids; others (urine screens)	Implemented detailed addiction assessment, separate treatment–planning template, dual–diagnosis groups, discharge referrals	45% of admissions misused substances but under–recognized in care plans and diagnoses	Odds of care–plan attention ×2.3; discharge Dx ×2.5; referrals ×1.8 after reforms	Low–cost protocol doubled identification and linkage to addiction care	Moderate
2	Greenfield et al., 1992 (USA) (44)	729 consecutive admissions to private inpatient SUD unit; 42 (5.80%) used drugs during stay	Inpatient substance–abuse treatment program in psychiatric hospital	Heroin/methadone, cocaine, marijuana; urine toxicology, observation, self–report	Supervised random urine screens ≥3/wk, staff vigilance, discharge on detection	On–ward use clustered in time; opioid dependence strongest risk factor	Comprehensive detection caught 85% events; heroin/methadone users OR≈3 for on–ward use	One relapse foretells others; routine broad toxicology essential	Low-moderate
3	Phillips & Johnson, 2003 (UK) (29)	264 psychosis in–patients across 11 wards; 127 (48.9%) dual–diagnosis	Acute general and PICU wards, 3 inner–London hospitals	Alcohol, cannabis, crack, opioids, others; AUS/DUS ratings + interviews	Policies + sanctions (leave withdrawal, discharge) & intermittent urine screens	83% of dual–diagnosis pts continued on–ward use; intimidation, dealing	Cannabis most frequently used on ward (52%)	Current controls insufficient; need proactive, therapeutic management	Low-moderate
4	Alterman et al., 1982 (USA) (28)	533 VA acute/sub–acute inpatients; 56 active drug users on ward (58% of drug–history pts)	Veterans acute & sub–acute psychiatric wards	Marijuana, amphetamines, alcohol, others; staff ratings	Nursing surveillance; ad–hoc responses	Negative attitudes, cliques, secretiveness; ↑supervision demand	10 recurrent adverse effects; drug users younger, more readmissions	Covert on–ward use common and disrupts care; structured policies needed	Low-moderate
5	Jegede et al., 2018 (USA) (31)	830 adult inpatients in community hospital psychiatry service	General adult acute psychiatric wards	Tobacco (52%), cannabis (32%), cocaine (23%), etc.; urine tox + chart review	Smoking status documented; 92% counseled, 64% offered NRT	Smoking strongly predicted multisubstance use, male sex, psychosis	Adjusted OR 3.1 for co–use among smokers	Systematic screen + cessation pharmacotherapy should be routine	Low-moderate
6	Wilson et al., 2010 (UK)	Trust–wide policy description; audit of all inpatient wards (figures not reported)	All acute mental–health & PICU wards, Manchester	Any illicit drugs & alcohol; routine screening criteria	Comprehensive policy: personal searches, confiscation, motivational/harm–reduction groups, staff training	Rise in substance–related untoward incidents prompted policy	Follow–up audits show fewer incidents & ↑staff clarity	Clear governance framework balances custodial & therapeutic priorities	Moderate
7	Cohen et al., 1999 (UK) (43)	Policy/clinical–governance analysis (no patient sample)	Psychiatric inpatient services nationally	Alcohol & drugs (general)	Calls for national guidelines, tiered staff training, systemic culture change	Milieu safety, legal & ethical dilemmas, duplication of local effort	Identified urgent need for strategic guidance	Substance misuse on wards is a governance challenge needing coordinated policy	Low-moderate
8	Berg & Restan, 2013 (Norway) (40)	Census day: 25 acute–ward residents; 8 (32%) had substance–induced psychosis; annual records n=807	36–bed acute psychiatric facility	Alcohol, cannabis, stimulants, benzodiazepines, etc.; medical records	Detox within ward; lack of dedicated emergency detox facilities	Reluctance to record SUD as primary; diagnostic & logistic challenges	Only 12.9% annual admissions coded with SUD primary despite high prevalence	Dedicated emergency detox beds or liaison model recommended	Low-moderate
9	Simpson et al., 2011 (UK)	Cross–sectional survey of 136 acute wards (3 shifts × 38,209 shift–reports)	Adult acute psychiatric wards across 100 NHS Trusts	Alcohol & illicit drug incidents recorded per shift	Exit security levels (open vs locked), breath–tests, drug dogs, searches	Use events rare (median 0) but staff concerned; unclear relationship to violence	No consistent association between locked doors and substance incidents	Active monitoring more useful than simply locking doors	Low-moderate
10	Graham et al., 2016 (UK)	59 inpatients with psychosis + SUD across 14 wards; pilot RCT BIMI vs TAU	Adult mental–health inpatient wards	Alcohol & drugs (self–report and SDS scale)	Brief Integrated Motivational Intervention (two 30–min sessions + workbook)	Low motivation & engagement with services	Engagement in community substance treatment at 3 mo: 52% BIMI vs 39% TAU (OR 1.63)	BIMI feasible; signals benefits for post–discharge engagement	Moderate
11	Yuodelis–Flores & Ries, 2008 (USA)	Chart review of substance–induced suicidal admissions to acute psych unit (no primary sample)	Urban acute psychiatric service	Alcohol, stimulants, opioids, etc.; toxicology + interviews	Integrated psychiatric + addiction management; discharge planning	High addiction severity; transient suicidality complicates placement	Detailed clinical characteristics and outcomes summarized	Need specific addiction interventions within psych emergency care	Low-moderate
12	Steinauer et al., 2017 (SWZ) (41)	329 admissions across 3 periods (pre/post door policy change)	Acute substance use and dual diagnosis ward	Alcohol, opioids, stimulants, cannabis, polysubstance	Shift from locked-door to primarily open-door policy; weekly random urine screens; breathalyzer; staff monitoring	Locked-door status used to prevent absconding, manage perceived risk of substance use; concern over ward safety and coercion	Significant 85% reduction in coercive measures; no significant change in substance use or violence; improved voluntary treatment rates	Open-door policy is feasible in acute SUD/dual diagnosis care and reduces coercion without increasing substance use or violence	Moderate
13	Ratschen, 2014 (UK)	Special article: narrative discussion (no primary sample)	Acute adult mental health inpatient wards	Tobacco; e–cigarettes (nicotine)	Advocates provision of e–cigarettes alongside NRT within smoke–free policy as harm–reduction tool	Entrenched smoking culture; policy enforcement challenges; health harms; staff time facilitating breaks	Synthesizes emerging evidence that e–cigs are far less hazardous and acceptable to patients	E–cigarettes could help psychiatric units achieve truly smoke–free status without exacerbating distress	Low-moderate
14	Barnett et al., 2021 (USA)	Correspondence: draws on literature (no primary sample)	General hospital inpatient wards (including psych units)	Illicit drugs (opioids, stimulants etc.)	Recommends Hospital Misuse Checklist, addiction consults, harm–reduction strategies, clear policies	In–hospital use common; risks overdose, infection, stigma; unclear staff response	Sets out practical recommendations for structured, compassionate management	Hospitals should adopt harm–reduction, patient–centered policies rather than punitive responses	Low
15	Searby et al., 2023 (Australia)	Scoping review of 20 studies on locked doors	Acute mental health inpatient units (mostly adult)	Illicit substances (door locking cited as contraband prevention)	Locked external doors intended to block drugs entering wards	No evidence doors reduce drug importation; may harm therapeutic relationship	Poor evidence for door locking; negative impact on staff satisfaction & consumer experience	Routine door locking lacks empirical support; alternative, least–restrictive risk management needed	High-moderate
16	Martin et al., 2023 (USA) (36)	Policy–development case example (no quantitative sample)	Safety–net teaching hospital acute & specialty wards	All substances (focus on opioids & smoking supplies)	Interprofessional revision of in–hospital substance use policy to remove security–first approach	Punitive responses led to arrests, stigma, patient harm, inequities	Updated policy emphasizes pain control, MOUD, support tools; issued 8 best–practice recommendations	Equity–oriented, non–punitive policies provide safer, more compassionate care for in–hospital substance use	Low-moderate
17	Johnson et al., 2020 (USA) (32)	942 adolescent inpatients (mean age 15.8); 158 SBIRT–eligible; staff interviews (n=14)	34–bed adolescent psychiatric inpatient unit	Alcohol and drugs (CRAFFT screening)	Motivational Enhancement–based SBIRT session (~60 min) delivered when possible	High SUD risk; brief LOS and staffing barriers limited intervention reach	Only 19% eligible teens received SBIRT; qualitative data identified workflow/staffing solutions	SBIRT is acceptable but needs dedicated resources or digital formats to reach more inpatients	Moderate
18	Kracher et al., 2023 (USA)	All admissions to 16–bed adult Brief Treatment Unit; ~88% had substance involvement	ED–diversion brief psychiatric inpatient unit	Alcohol & illicit drugs (AUDIT, DAST)	Nurse–administered SBIRT after staff training & coaching	Substance use drove high readmissions & recidivism	SBIRT offered to 59% admissions; readmissions fell 18% overall & 68% at 16–31 days	Frontline nurse–led SBIRT can meaningfully cut psychiatric readmissions within weeks	Moderate

### 3.2 Main themes

**Figure 2:**
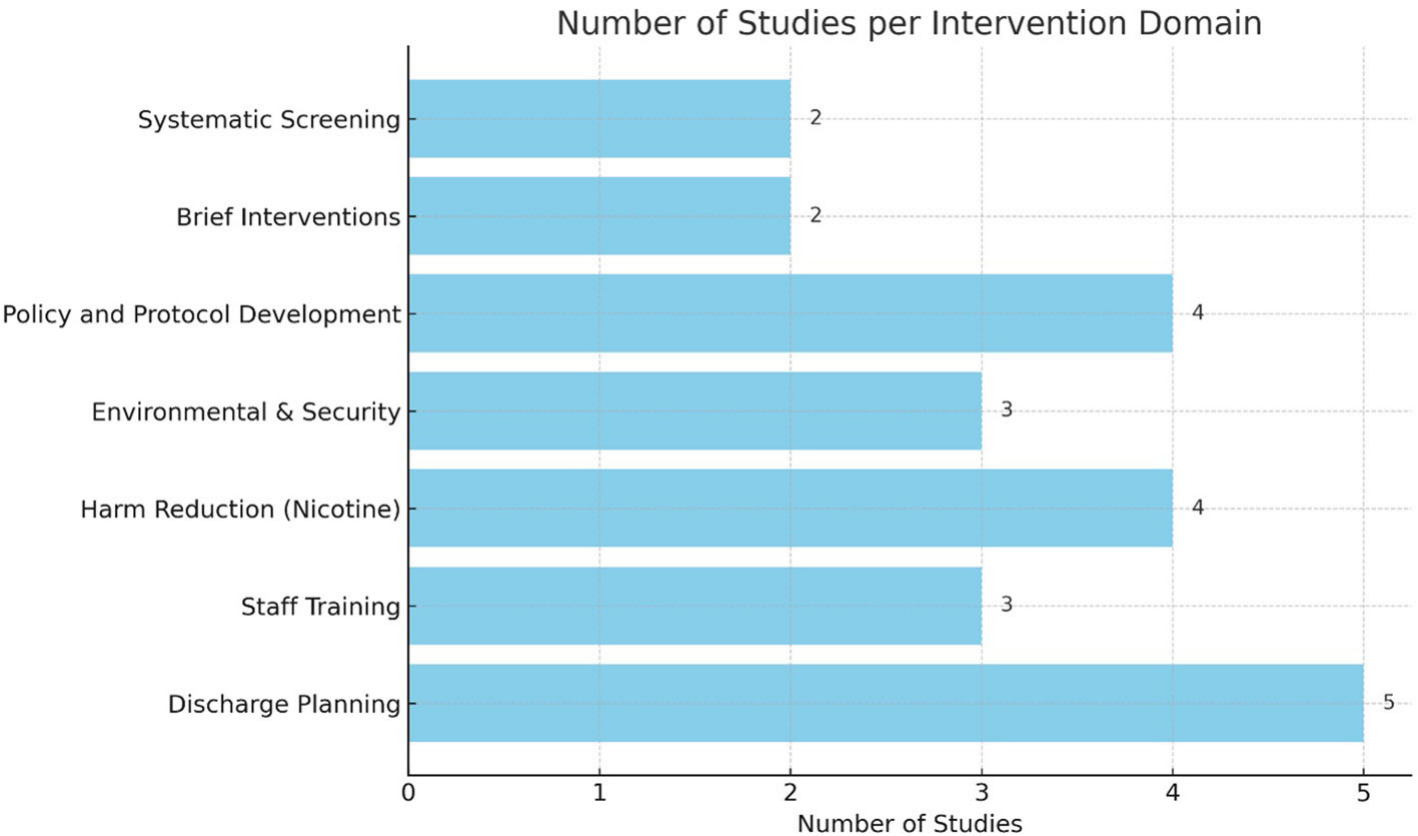
Number of studies identified per intervention domain.

Seven themes, mapped as intervention domains, were identified across the 18 studies. **Figure 2** outlines the number of studies found for each intervention domain. Each domain will be presented underneath to understand its key points and potential use and benefits.

#### 3.2.1 Systematic screening & identification

A consistent message across the identified studies is that effective management begins with universal, structured case-finding at (and throughout) admission. Older prevalence studies (e.g., Alterman et al., 1982; Phillips et al., 2003) relied on clinician judgment and *ad-hoc* urine testing, but more recent work shows the benefit of formal tools such as the Alcohol Use Disorders Identification Test (AUDIT), Drug Abuse Screening Test (DAST), CRAFFT Screening Test for Adolescent Substance Use (CRAFFT), or single-question screeners embedded in the electronic chart (28, 29). Prochaska and colleagues demonstrated that adding a brief, algorithm-driven intake checklist doubled the proportion of in-patients whose substance use was recognized in care plans and discharge documents, while Jegede showed that routine urine toxicology uncovered polysubstance use in more than half of admissions (information that would otherwise have been missed) (30, 31). Adolescent services face unique challenges: Johnson et al. found that CRAFFT screening identified 158 at-risk youths in a single year, but time constraints meant that only one in five ultimately received an intervention, underscoring the need for streamlined digital or nurse-led workflows (32). At a systems level, the Joint Commission’s Hospital Based Inpatient Psychiatric Services (HBIPS-1) quality indicator now requires documented substance-use screening on every U.S. psychiatric admission, providing both an incentive and a benchmark for compliance monitoring. Together, these findings argue for standardized, auditable screening pathways, ideally hard-wired into admission orders and repeated after periods of leave or changes in mental state, to convert detection from a discretionary act into a routine standard of care.

#### 3.2.2 Brief interventions & integrated therapeutic care

Once substance use is detected, the evidence favors brief, motivationally oriented interventions delivered on the ward and linked to post-discharge care. The pilot RCT by Graham et al. showed that a two-session Brief Integrated Motivational Intervention (BIMI) increased engagement with community addiction services from 39% to 52% three months post-discharge (33). In parallel, nurse-administered SBIRT programs have gained traction: Kracher et al. reported SBIRT uptake in 59% of admissions and an 18% reduction in readmissions within a month, while Johnson et al. confirmed its feasibility in adolescents despite staffing hurdles (32, 34). These programs are most effective when embedded in a broader dual-diagnosis model. For example, Prochaska et al., paired structured assessment with daily group therapy and dedicated discharge planning, doubling documentation of substance-related goals (30). The common elements across successful initiatives include: (a) a manualized, 10- to 60-minute counseling protocol grounded in motivational interviewing; (b) rapid referral pathways to community or hospital-based addiction specialists; and (c) routine outcome tracking (e.g., readmissions, engagement rates) to drive quality-improvement cycles. Ward teams therefore benefit from adopting a tiered intervention ladder, starting with bedside brief advice for low-risk users and scaling up to specialist addiction liaison for complex dependence.

#### 3.2.3 Policy & governance frameworks

Empirical work and practice commentaries converge on the idea that clear, equity-oriented written policies are the backbone of sustainable substance-use management. Wilson 2010’s trust-wide guideline combined motivational approaches with explicit search and confiscation procedures, resulting in fewer substance-related incidents and improved staff confidence (35). Martin extended this by rewriting a hospital policy that had relied on security-led enforcement; the new version centered on harm-reduction, medication for opioid-use disorder (MOUD), and staff discretion grounded in trauma-informed care (36). At a national level, NICE Guideline NG58 mandates integrated care planning for co-existing severe mental illness and substance misuse, offering a template that units can adapt (16). Studies emphasize that governance goes beyond paperwork: Williams and Cohen showed that pairing policies with audit-feedback loops (tracking screening rates, incident reports, and patient complaints) creates a virtuous cycle of accountability (37). Importantly, policies should define graduated responses (from supportive counseling to, rarely, legal action) and outline staff roles, documentation standards, and escalation pathways. Where policies are co-produced with patients and community partners, units report greater legitimacy and fewer adversarial interactions, highlighting governance as both a procedural and relational endeavor.

#### 3.2.4 Environmental & security measures

Physical controls remain the most controversial domain. Large multi-ward surveys and the recent scoping review by Searby et al. find no consistent link between locked external doors and reduced on-ward substance use; in fact, door locking may erode therapeutic alliance and staff morale (38, 39). Targeted measures (random breath-tests, urine toxicology, or passive drug-detection dogs) can detect contraband but risk a punitive tone if not paired with rapid therapeutic follow-up. Alterman documented how covert on-ward use created patient cliques and supervision burdens (28). However, Berg and colleagues showed that simply adding detox beds reduced illicit consumption by providing a safe, sanctioned space for withdrawal (40). The emerging consensus favors a least-restrictive, risk-responsive model: searches based on reasonable suspicion rather than blanket policies, locked doors used sparingly and reviewed daily, and environmental cues (visibility lines, staff presence) designed to dissuade dealing without creating a carceral atmosphere. Units implementing such nuanced strategies report stable incident rates, lower seclusion use, and better patient satisfaction, suggesting that environmental measures work best when they support (not substitute for) therapeutic engagement. Similarly, Steinauer and colleagues reported that shifting from locked to primarily open doors on a substance use and dual diagnosis ward led to a significant 85% reduction in coercive measures, without any increase in substance use or violence, further challenging assumptions that door-locking policies are necessary for safety or substance control (41).

#### 3.2.5 Harm-reduction

An increasing number of inpatient units are adopting harm-reduction approaches for substances other than tobacco. For example, Martin details protocols that provide take-home naloxone, initiate MOUD during admission, and allow patients to store vaping devices (36). Barnett et al. proposes safe-use kits and no-punish frameworks for patients who disclose in-hospital drug use, arguing that punitive responses drive concealment and overdose risk (42). On the alcohol front, some units have implemented symptom-triggered benzodiazepine tapers and monitored consumption agreements rather than zero-tolerance bans, reporting fewer withdrawal complications. These initiatives seek to mitigate risks associated with ongoing substance use during hospitalization, aligning inpatient practice with broader public-health trends in harm reduction.

#### 3.2.6 Staff training & culture change

Technical protocols are difficult to apply without a workforce that is both skilled and philosophically aligned with integrated care. Early ethnographic work revealed cultures of “us versus them,” where substance-using patients were judged more than helped (28, 43). Contemporary programs address this through multidisciplinary training, reflective practice, and ongoing coaching. Wilson and colleagues embedded an annual competency package covering motivational interviewing, trauma-informed search techniques, and de-escalation; staff surveys documented improved confidence and a 20% drop in incident reports (35). Kracher et al. paired SBIRT roll-out with bedside mentorship, finding that sustained coaching, rather than one-off workshops, predicted intervention fidelity (34). Senior-management ownership and peer champions are repeatedly cited as catalysts for shifting norms from rule enforcement to therapeutic alliance. Measuring culture is equally important: many trusts now incorporate staff attitudes toward dual diagnosis into routine dashboards, signaling that culture change, like any outcome, is audited, resourced, and rewarded.

#### 3.2.7 Discharge planning & continuity of care

Inpatient success is fragile without seamless hand-off to community addiction services. Prochaska et al. and Graham et al. (both link structured discharge plans (and explicit substance-use referrals) to higher engagement rates and lower early readmissions (30, 33). Greenfield et al. showed that detecting on-ward relapse, coupled with a clear aftercare contract, mitigated the clustering of post-discharge use episodes (44). Nurse-led SBIRT models of Kracher et al. now include warm hand-offs to peer recovery coaches and digital reminders, achieving up to a 68% reduction in 16–31-day readmissions (34). Effective plans share three traits (1): documented linkage (appointment date/time or e-referral confirmation) (2), patient-centered goal-setting that integrates mental-health and addiction priorities, and (3) follow-up accountability, whether by community teams, virtual check-ins, or text-based support. Emerging digital solutions (secure messaging, tele-SBIRT) show promise for bridging the important first two weeks after discharge, a window repeatedly flagged as high-risk for relapse or overdose. Embedding these elements transforms discharge from an administrative act into a continuum of care that maintains therapeutic momentum beyond the ward.

### 3.3 Quality appraisal

Using the JBI suite of critical-appraisal tools matched to each study, the overall evidence base is moderate. The lone randomized study of Graham et al. met seven of thirteen RCT criteria, earning moderate quality: random-sequence generation and baseline comparability were clear, but allocation concealment, blinding, and power calculations were absent, and follow-up was under 80% (33). The three quasi-experimental or quality-improvement reports scored between six and eight on the nine-item checklist, also achieving a moderate rating: they provided clearly defined interventions, multiple pre-post measures, and parallel controls or statistical adjustments, but relied on convenience samples and were susceptible to history effects (30–32, 34). Among the seven descriptive prevalence or cross-sectional studies, half satisfied six to eight JBI reporting standardized measures, transparent inclusion criteria, and more than 80% participation, while the remainder lacked probability sampling or had substantial missing data, yielding low-to-moderate ratings (28, 29, 31, 38, 40, 44, 45). The two policy/governance case studies and the Lancet Psychiatry commentary were appraised with JBI’s “text and opinion” checklist: both policy papers articulated clear positional statements supported by referenced evidence and stakeholder consultation (scoring 4/6, moderate), whereas the commentary was more impressionistic (3/6, low) (35, 36, 42). The Searby et al. scoping review scored nine out of eleven criteria on the JBI comprehensive search strategy checklist, duplicate selection, and transparent charting, but did not register a protocol or appraise included evidence, so quality was classed as high-moderate (39). Finally, the earlier governance met only three out of the six applicable criteria (unclear search methods, unstructured synthesis), thus low quality (43). In sum, about one-third of the identified study are moderate-to-high quality. As a general appraisal, the field is limited by small samples, single-site designs, and often descriptive aims.

## 4 Discussion

### 4.1 Findings and comparison with prior works

The present scoping review identified 18 empirical and practice-oriented publications that describe how psychiatric wards attempt to detect, manage, or otherwise respond when patients consume alcohol or drugs during admission. Most studies originated from high-income countries and focused on general adult units; only a handful examined adolescent, forensic, or brief-stay settings. Seven broad management domains were identified: systematic screening, brief motivational care, policy and governance frameworks, environmental or security measures, harm-reduction initiatives, staff training and culture change, and discharge linkage. Many studies are descriptive, small-scale, or limited to single-site quality improvement efforts, with few rigorous trials or multi-site evaluations. This gap is particularly striking given the contrast between the prevalence of substance use in psychiatric settings and the lack of robust guidance for clinicians. Bridging this gap will require more systematic research efforts, drawing on both quantitative and qualitative methods to inform pragmatic, patient-centered approaches to care. Nevertheless, consistent signals emerged: structured screening improves detection, brief interventions are feasible, and purely punitive security tactics rarely prevents contraband entry or aggression.

One notable finding is the underutilization of structured screening tools in psychiatric settings. While nurse-led screening has proven effective in general hospitals (identifying unhealthy substance use in approximately 16% of patients), psychiatric units often lack standardized protocols (46). Implementing brief screening tools, such as the AUDIT, into routine assessments could enhance early detection and intervention efforts. Harm-reduction approaches, have also shown promise in mental health settings. As an example, despite the implementation of smoke-free policies, tobacco use remains highly prevalent on psychiatric wards, often leading to daily tensions, withdrawal symptoms, and patient agitation due to inconsistent enforcement and limited alternatives. Providing NRT and considering the supervised use of e-cigarettes can help manage withdrawal symptoms during hospitalization, reduce conflict on the ward, and support both patient comfort and adherence to smoke-free regulations (47). More broadly, staff attitudes toward patients with substance use disorders significantly influence care quality. Educational interventions have demonstrated effectiveness in improving nurses’ attitudes and perceptions, leading to more compassionate and effective care (48). Ongoing training and support for healthcare providers are essential to favorize a therapeutic environment conducive to recovery.

Harm-reduction approaches represent a particularly important yet underdeveloped area in this literature. Several studies describe promising ward-level strategies (including safe-use kits, monitored alcohol tapering, naloxone distribution, and permissive frameworks for disclosure) that help mitigate the risks of ongoing substance use during hospitalization (36, 42). However, such practices remain inconsistently implemented and are often hampered by prevailing abstinence-based or punitive ward cultures. A recurring theme across the studies reviewed is that harm reduction is not simply a set of protocols but a shift in clinical mindset: one that requires sustained investment in staff training, ongoing coaching, and reflective practice to be effective. One-time educational sessions alone appear insufficient to embed harm-reduction principles into daily care. Favorizing a culture of compassionate, risk-mitigating engagement (particularly in the face of entrenched stigma toward substance use) is important for improving patient outcomes and bridging the current evidence-practice gap in psychiatric inpatient settings.

From a policy perspective, the findings underscore the need for national and regional mental health frameworks to explicitly address substance use occurring within psychiatric inpatient settings, rather than focusing solely on discharge planning. Incorporating ward-level protocols into accreditation standards, hospital governance audits, and quality indicators could incentivize adoption of evidence-informed strategies. Policies that integrate harm-reduction principles and ensure funding for staff training and screening infrastructure are more likely to achieve sustainable impact. Finally, practical barriers reported included limited staffing, high patient acuity, competing clinical priorities, and restricted ward environments that limit privacy for interventions. Resistance from staff unfamiliar with harm-reduction principles and lack of leadership endorsement were also recurrent challenges. Implementation was more successful where interventions were embedded into routine workflows, supported by leadership, and accompanied by ongoing mentorship rather than one-off training sessions.

### 4.2 Limitations

This review is not without limitations. Only English- and French-language sources were included, and grey literature beyond peer-reviewed journals was not systematically searched, raising the possibility that effective local protocols remain unpublished. The evidence base itself is skewed toward descriptive reports with small samples; many lacked control groups, standardized outcome measures, or long-term follow-up, limiting confidence in causal inferences. Finally, heterogeneity in unit type, patient mix, and health-system context hindered direct comparison of interventions. These gaps underscore the need for multi-site implementation studies and consensus-building exercises that can translate promising but disparate strategies into coherent, evidence-informed guidance for psychiatric inpatient care. A limitation of the current evidence base is the absence of standardized outcome measures. Across studies, outcomes ranged from detection rates and patient engagement to qualitative staff perceptions, making cross-study comparison difficult. Future work would benefit from a core outcome set for inpatient substance-use interventions, encompassing both clinical (e.g., relapse, readmissions) and process measures (e.g., screening adherence, patient satisfaction). Such standardization would facilitate meta-analyses and guide quality-improvement benchmarks.

### 4.3 Recommendations

This scoping review highlights several areas where practical, evidence-informed improvements can be made to better manage substance use on psychiatric inpatient units. Recommendations are summarized in **Table 2**.

**Table 2:** Summary of recommendations.

Intervention domain	Key recommendation	Supporting studies
Systematic Screening	Implement standardized screening (e.g., AUDIT, DAST) at admission to identify substance use early.	Prochaska et al. (2005); Johnson et al. (2020) (30, 32)
Brief Interventions	Use adapted SBIRT or motivational interviewing approaches feasible for psychiatric wards.	Graham et al. (2016); Johnson et al. (2020) (32, 33)
Policy and Protocol Development	Develop clear, patient-centered protocols for handling on-ward substance use, intoxication, and contraband.	Wilson et al. (2010); Martin et al. (2023) (36)
Harm Reduction	Implement safe-use practices (e.g., take-home naloxone, monitored alcohol tapering, non-punitive disclosure frameworks) to mitigate risks associated with ongoing substance use during hospitalization.	Raschen et al. (2009); Jegede et al. (2018) (31)
Staff Training and Consistency	Offer regular training in dual diagnosis, trauma-informed care, and structured de-escalation.	Jegede et al. (2018); Prochaska et al. (2005) (30, 31)

1. The implementation of systematic screening procedures is both feasible and necessary. Several included studies, such as Prochaska et al. and Johnson et al. demonstrated that embedding brief screening tools like AUDIT and DAST at admission can identify substance use early and create an opportunity for timely intervention (30, 32). Where staffing allows, universal screening should be incorporated into routine nursing or medical assessments.
2. Brief motivational interventions tailored to psychiatric inpatients should be expanded. Interventions such as SBIRT, adapted for the ward environment, showed promising acceptability and early impact in adolescent and adult settings (32, 33). Although implementation challenges were reported (such as patient acuity and staff training needs) these approaches were found to be both low-cost and scalable when properly supported.
3. Third, there is a need for clear and consistently applied ward-level policies. Studies underscored the importance of having written protocols for managing suspected on-ward substance use, including guidance on searches, use of breathalyzers, and steps for managing intoxication (35, 36). These policies should be transparent, patient-centered, and include graduated responses rather than purely punitive approaches.
4. In line with harm-reduction principles, several reports recommend the provision of nicotine-replacement therapy and consideration of alternatives such as e-cigarettes in smoke-free psychiatric facilities (31, 45). These measures were associated with improved patient adherence to smoking bans and reduced conflict. While full harm-reduction programs for other substances are not yet widely implemented on inpatient units, these studies highlight a direction for future practice.
5. Finally, ongoing staff education and reflective practice are important to ensure consistent, stigma-free responses to substance use on the ward. Jegede et al. and Prochaska et al. both noted wide variability in how staff respond to substance-related incidents, with some staff expressing uncertainty or moral discomfort (30, 31). Regular training on dual diagnosis, trauma-informed care, and structured de-escalation can support therapeutic consistency and reduce reliance on containment measures. Incorporating principles of culturally safe care and cultural humility is also important, as substance use patterns, stigma, and treatment expectations can vary considerably across cultural groups. Attention to these dimensions can help tailor interventions, favorize trust, and improve engagement among diverse patient populations. Cultural context strongly shapes substance-use patterns, help-seeking behaviors, and perceptions of harm-reduction practices. Adapting interventions for cultural relevance (through co-design with service users, inclusion of culturally specific peer workers, and integration of Indigenous or community-based healing approaches) can improve engagement and trust. This is particularly important in diverse urban psychiatric settings and for Indigenous and minority populations, where historical mistrust of psychiatric institutions may affect intervention uptake.

Resource considerations are very important to implementation. Most strategies identified (such as brief motivational interventions, standardized screening tools, and staff training) are relatively low-cost when integrated into existing workflows, but require initial investment in training and change management. More resource-intensive measures, such as dedicated addiction liaison staff or environmental modifications, may be justified in high-prevalence settings but should be evaluated for cost-effectiveness.

## 5 Conclusion

This scoping review provides a comprehensive synthesis of the limited body of literature on how psychiatric inpatient units manage substance use that occurs during hospitalization. Although practices vary considerably across settings, the findings reveal several promising strategies. Such strategies include structured screening, brief motivational interventions, clear ward policies, harm-reduction tools, and staff education. These strategies can be feasibly adapted to psychiatric contexts. When implemented thoughtfully, they have the potential to improve detection, enhance therapeutic engagement, reduce conflict, and promote continuity of care after discharge. However, the evidence remains fragmented, methodologically heterogeneous, and heavily reliant on single-site or descriptive reports. Moving forward, there is a pressing need for multi-site implementation studies, consensus-driven protocols, and pragmatic evaluations that reflect the complex realities of psychiatric wards. Strengthening the evidence base in this area will be essential not only to support frontline clinicians but also to help shift away from punitive or control-based responses that risk undermining trust and recovery. Embedding compassionate, harm-reduction-oriented practices into inpatient care is a critical step toward greater alignment with contemporary public health and addiction treatment frameworks. Future research should prioritize multi-site pragmatic trials and hybrid implementation-effectiveness studies that assess both clinical outcomes and contextual determinants of success. Longitudinal designs with standardized outcome sets, inclusion of diverse cultural groups, and cost-effectiveness analyses will be essential. Mixed-methods approaches can capture both quantitative impact and qualitative insights into patient and staff experiences, informing scalable and context-sensitive protocols.
